# Understanding and drugging RAS: 40 years to break the tip of the iceberg

**DOI:** 10.1242/dmm.049519

**Published:** 2022-03-04

**Authors:** Donita C. Brady, Julija Hmeljak, Arvin C. Dar

**Affiliations:** 1 Department of Cancer Biology, Perelman School of Medicine, University of Pennsylvania, Philadelphia, PA 19104, USA; 2 Abramson Family Cancer Research Institute, Perelman School of Medicine, University of Pennsylvania, Philadelphia, PA 19104, USA; 3 The Company of Biologists, Bidder Building, Station Road, Histon, Cambridge CB24 9LF, UK; 4 Icahn School of Medicine at Mount Sinai, Tisch Cancer Institute, Center for Therapeutic Discovery, Mount Sinai, New York, NY 10029-5674, USA

**Keywords:** Cancer, Developmental disorders, RAS inhibitor, RAS pathway

## Abstract

Several cancers and rare genetic diseases are caused by dysregulation in the RAS signaling pathway. RAS proteins serve as molecular switches that regulate pathways involved in cellular growth, differentiation and survival. These pathways have been an intense area of investigation for four decades, since the initial identification of somatic RAS mutations linked to human cancers. In the past few years, inhibitors against several RAS effectors, as well as direct inhibitors of the K-RAS mutant G12C, have been developed. This Special Issue in DMM includes original Research articles on RAS-driven cancers and RASopathies. The articles provide insights into mechanisms and biomarkers, and evaluate therapeutic targets. Several articles also present new disease models, whereas others describe technologies or approaches to evaluate the function of RAS *in vivo*. The collection also includes a series of Review articles on RAS biology and translational aspects of defining and treating RAS-driven diseases. In this Editorial, we summarize this collection and discuss the potential impact of the articles within this evolving area of research. We also identify areas of growth and possible future developments.

## Abstract (precis)

**Summary:** This Editorial introduces DMM’s new Special Issue on the RAS pathway. The Guest Editors reflect on the impact of the featured articles on the landscape of the RAS field.

## Introduction

The RAS superfamily of small GTPases includes over 150 members in humans (Wennerberg et al., 2005). These proteins generally function as molecular switches and regulators of cellular communication, linking cues from the cell surface to changes in gene expression and protein translation (Pylayeva-Gupta et al., 2011). The four RAS oncoproteins, K-RAS4A, K-RAS4B, N-RAS and H-RAS, encoded by three RAS genes, are the founding members of the family and recognized for their critical roles in cancer (Moore et al., 2020). Indeed, one in four human cancers contain mutant forms of RAS, with substitutions frequently observed in pancreatic cancer (∼88% K-RAS mutation positive), colon cancer (∼50% K-RAS), lung cancer (∼32% K-RAS) and melanoma (∼17% N-RAS) (Prior et al., 2020). These key members, together with additional RAS homologs, have been implicated in other diseases, including common Mendelian disorders referred to as RASopathies (Simanshu et al., 2017), non-neoplastic cerebral diseases, such as Alzheimer's and Parkinson's disease (Qu et al., 2019), and spinocerebeller ataxia type I (Park et al., 2013).

2022 marks the 40th anniversary of the initial discovery of RAS mutations in human cancers (Der et al., 1982; Parada et al., 1982; Santos et al., 1982). Over this time, RAS family members – in particular K-RAS due to the prevalence of the mutants – have been intensively studied and have captivated scientists from a range of disciplines, including structural biology, biochemistry, chemistry, signal transduction, model organism development, genomics and medicine, among others. A watershed moment occurred with the first discovery of direct K-RAS inhibitors targeting the G12C mutant through a covalent mechanism (Ostrem et al., 2013). Recent clinical testing of the mechanism of these inhibitors, including adagrasib and sotarasib (Kim et al., 2020), has demonstrated significant clinical responses (Hong et al., 2020; Riely et al., 2021; Sabari et al., 2021).

**Fig. 1.**
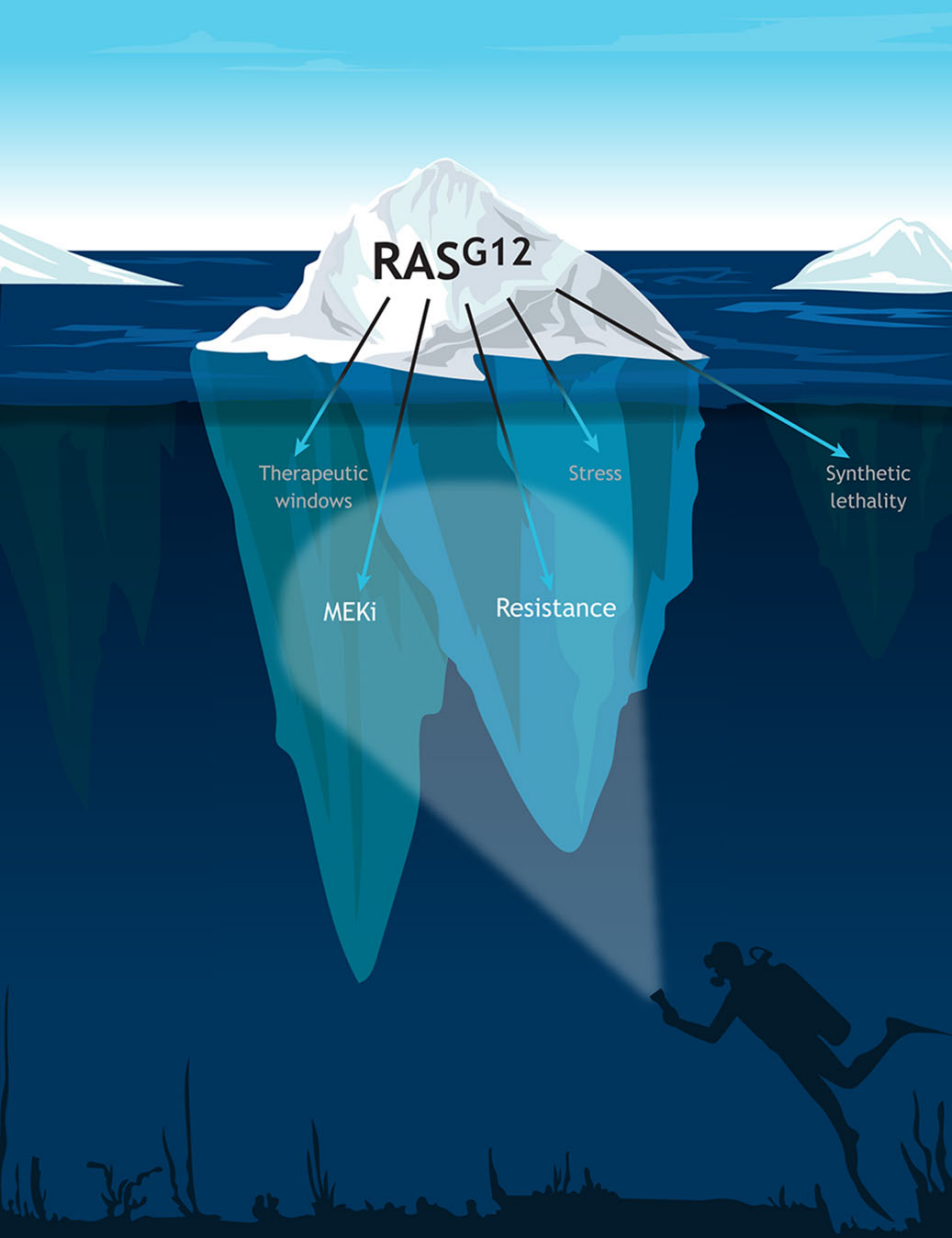
**The RAS pathway beyond the tip of the iceberg.** Over the past four decades, researchers have encountered successes and hurdles in effective targeting of aberrant RAS signaling downstream of the touchstone RAS^G12^ mutation. Although important progress has been made with regards to MEK (MAP2K) inhibitors (MEKi) and approaches to overcome acquired resistance, future research will need to focus on less explored areas, such as cellular stress, novel therapeutic windows within the pathway itself and synthetic lethality.

The story of RAS from human genetics through to the first direct inhibitors represents a long and winding road of discovery, which has been greatly accelerated by a focused community of academic and industry scientists (Nissley and McCormick, 2022). However, we are likely only at the tip of the iceberg for understanding RAS (Fig. 1). Under the surface, much remains to be understood about different RAS family members and about targeting the dysregulated pathways with therapeutic interventions. This Special Issue of Disease Models & Mechanisms (DMM) was thus conceived to collate cutting-edge research on the roles of RAS in cancer and developmental disorders, and on approaches to treat and modify the disease course in model systems. In this Editorial, we reflect on this Special Issue and how its articles fit into the broader context of this exciting field.

**Figure:**
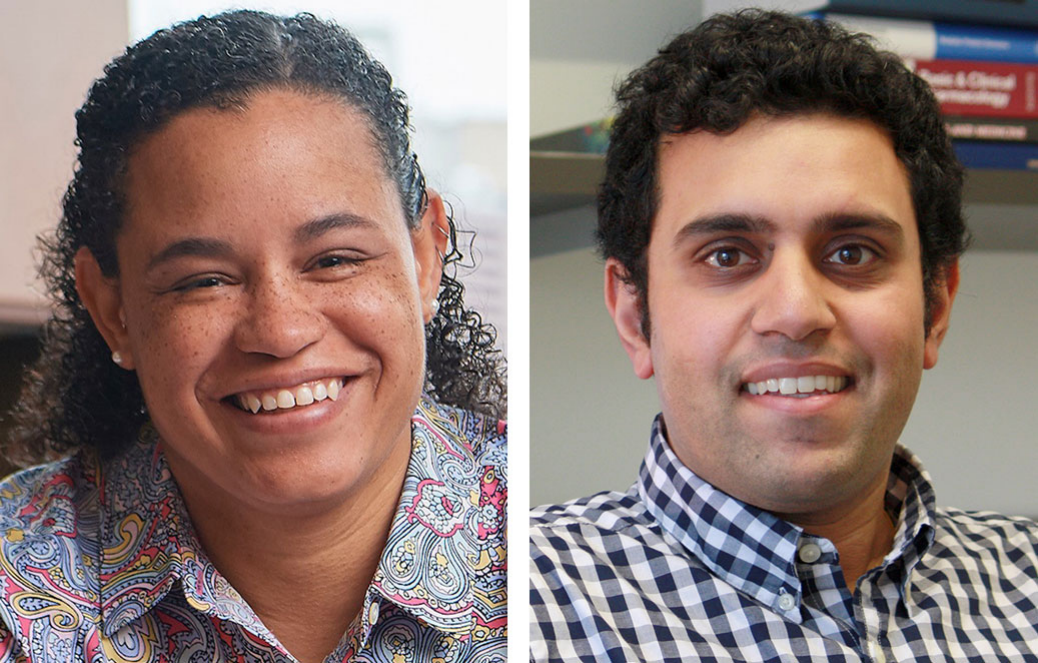
Donita Brady (left) and Arvin Dar (right)

## RAS targeting in cancer

Aberrant activation of the RAS pathway is the most common driver in human cancers. In the four decades since the discovery of RAS mutations in cancer, researchers have made significant advances in understanding their mechanistic underpinnings. For example, work previously published in DMM has shown that dysregulated RAS signaling affects phenotypic plasticity (Bangs et al., 2020; López-Cuevas et al., 2021), an emerging hallmark of cancer (Hanahan, 2022). Several such interdisciplinary studies focus on the oncogenic output of dysregulated RAS signaling and provide essential bases for identifying actionable therapeutics. In this Special Issue, a new Review article from Julien Ablain's group analyses how RAS dysregulation in melanoma is a paradigm for successful translation of mechanistic insight into targeted therapies for cancer patients (Al Mahi and Ablain, 2022). Expanding on this, a Review by Elda Grabocka and colleagues explores the relationships between RAS signaling and stress response pathways, and discusses how these present novel therapeutic windows for RAS-driven cancers (Redding et al., 2022). Along these lines, Zhe Han and colleagues report a *Drosophila* model for acute myeloid leukemia driven by K-RAS^G12V^ and identify several hypoxia pathway genes as possible drug targets. They also show the efficacy of the HIF-1α inhibitor echinomycin in these models (Zhu et al., 2021). Similarly, Catrin Pritchard's group demonstrate that statins inhibit early-stage BRAF^V600E^-driven lung tumors by altering the immune microenvironment (Kamata et al., 2021). Together, these findings confirm that targeting cellular and microenvironmental stress pathways is indeed a viable therapeutic opportunity.

A challenge in the RAS field has been understanding the function of RAS in membranes. Marcel Schaaf and colleagues use single-molecule microscopy in a zebrafish model to study the dynamics and mobility of wild-type and mutant H-RAS. Based on their observations, the authors relate membrane dynamics to the activation state of RAS (Gora et al., 2022). Aside from shedding light on fundamental functions, model organisms can also reveal unexpected pathways or targets for therapeutic intervention. Jason Yustein's group reports a genetically engineered mouse model of rhabdomyosarcoma (RMS) (Nakahata et al., 2022). This model faithfully recapitulates the tumor histology in RMS patients, and overcomes some caveats from previously developed models of RMS. Lastly, Nilsson and colleagues report a new mouse model of sporadic thyroid carcinoma generated through inducible expression of BRAF^V600E^ (Schoultz et al., 2021). The *in vivo* RAS-driven cancer models in this issue provide new tools to evaluate therapeutics and to identify new targets for intervention.

## The RASopathies

Germline mutations in genes encoding components of the RAS pathway cause a group of rare developmental disorders called the RASopathies. Improved understanding of the mechanisms of RAS dysregulation, coupled with expanded clinical sequencing means that the definition of RASopathy continues to evolve, as discussed in Katherine Rauen's Perspective (Rauen, 2022). This article explores the enigma of defining a RASopathy through the lens of oncologists, medical geneticists, RAS biologists, and patients and their families. This holistic approach aims to advance our understanding of these developmental disorders and how they can be diagnosed and treated. Research in RAS dysregulation, both in the context of cancer and of RASopathies, has identified several potential therapies for RASopathy patients, as discussed in the Special article by Corina Anastasaki and David Gutmann (Anastasaki and Gutmann, 2022) and in the Review from Marielle Yohe and team (Hebron et al., 2022). Indeed, a research article from Katherine Rauen's group describes a newly developed murine model of the RASopathy Costello syndrome and demonstrates that MEK inhibition is an effective strategy in mitigating the muscular damage caused by aberrant H-RAS (Tidyman et al., 2021).

Whilst RASopathies are developmental disorders driven by mutations in components of the RAS pathway (Rauen, 2022), the involvement of aberrant RAS signaling in development reaches beyond this group of disorders. Toshihiro Inubushi and colleagues found that a transcriptional regulation pathway downstream of RAS is required in normal palatogenesis and its perturbation may be involved in cleft palate (Inubushi et al., 2022). A new mouse model was also instrumental in understanding the role of sensory neuron genesis and differentiation in the neurodevelopmental disorder DiGeorge (also known as 22q11.2 deletion) syndrome (Karpinski et al., 2021), which is caused by broad transcriptional deregulation affecting RAS signaling among other crucial pathways. Taken together, the RASopathies-focused articles in this Special Issue highlight recent progress in the field, which is already bringing tangible benefits to patients.

## Voices beyond this Special Issue

In this Special Issue, DMM editor Ross Cagan interviewed Kevan Shokat to discuss his success in ‘drugging the undruggable’ RAS and how blurring the boundaries between chemistry and biology can help develop novel cancer therapeutics (Cagan and Shokat, 2022). In her ‘A Model for Life’ interview, Shiva Malek talks about her career, personal attitudes towards mentorship, and how targeting RAS-driven cancers requires strong collaboration between academia and industry (Malek, 2021).

These conversations with leaders in the RAS field show how knowledge has evolved quickly in recent years, most notably with the introduction of novel drugs to enable translational studies. K-RAS^G12C^ was initially tractable due to the unique reactivity of the cysteine residue and the ability to find compounds that covalently target the mutant (Ostrem et al., 2013). Extending these approaches to other RAS mutants is currently underway, and it appears that the K-RAS^G12D^ mutant is also amenable to direct inhibition (Wang et al., 2021). In parallel, clinical studies have begun to report mechanisms of acquired resistance to K-RAS^G12C^ inhibition (Awad et al., 2021; Tanaka et al., 2021; Zhao et al., 2021). Similarly to studies on the rapid emergence of resistance against BRAF^V600E^ inhibitors (Swanton et al., 2014), this work highlights the inherent challenges of targeting single alleles. Common mechanisms to evade inhibitors, including mutations directly in the drug-binding pocket, overexpression of the oncogene and alterations in various effector pathways, mean that successfully targeting aberrant RAS signaling is an ongoing task. There have been several combination strategies with inhibitors of targets that function upstream, like SHP2 (PTPN11) (Fedele et al., 2021; Ryan et al., 2020) and EGFR (Amodio et al., 2020; Xue et al., 2020), or downstream of RAS (Adachi et al., 2020; Lou et al., 2019; Misale et al., 2019; Molina-Arcas et al., 2019; Santana-Codina et al., 2020). Additionally, combinations based on targeting immune-modulatory pathways (Briere et al., 2021; Canon et al., 2019) are under investigation. These efforts hope to expand the number of patients that benefit from direct KRAS inhibitors, as well as extend the duration of responses.

In addition to the translational work, recent years have brought further insight into the basic biology of RAS. For example, researchers have made major advances in our understanding of the structural biology of complexes, drug-binding mechanisms, and the regulation of distinct target activation states (Kondo et al., 2019; Liau et al., 2020; Martinez Fiesco et al., 2022; Park et al., 2019). Moreover, fundamental studies on how various mutants operate and why there could be strong lineage dependence for individual alleles is another exciting area of research. We anticipate that such insights focused on understanding RAS biology in cancer and RASopathies will have broad implications for many classes of diseases. Along such lines, targeting of the RAS pathway in diseases of aging (Fabian et al., 2021; Slack et al., 2015) and neurological disorders (Park et al., 2013; Qu et al., 2019) with anti-cancer therapeutics has already demonstrated promise for advances in these areas.

## Conclusions

The articles in and beyond this Special Issue highlight significant advances in the field, from improved understanding of aberrant RAS signaling to therapy development. The increasing amount and validity of novel cell and animal models, even for some of the rarest RASopathies and RAS-driven cancers, will allow for further characterization of the disease pathology and can facilitate the development of novel therapeutic interventions. Recognizing the overlap between RASopathies, aging, neurological disorders and RAS-driven cancer shows how enhancing knowledge of each of these diseases can be mutually beneficial. It has also become clear that standardizing outcome measures and validating findings are of utmost importance for effective translation from model systems to patients. This rigor can propel the RAS field forward and result in better selection of candidate compounds and their most effective combination strategies to target multiple levels of the signaling pathway, eventually leading to a higher success rate in clinical trials for the broad range of diseases that are linked to RAS. We hope that you enjoy this Special Issue and the ongoing subject collection, which will continue to grow as new articles on the topic are published in DMM.
